# Correction: *Effect of iron and zinc-biofortified pearl millet consumption on growth and immune competence in children aged 12–18 months in India: study protocol for a randomised controlled trial*

**DOI:** 10.1136/bmjopen-2017-017631corr2

**Published:** 2018-10-02

Mehta S, Finkelstein JL, Venkatramanan S, *et al*. Effect of iron and zinc-biofortified pearl millet consumption on growth and immune competence in children aged 12–18 months in India: study protocol for a randomised controlled trial. *BMJ Open* 2017;7:e017631. doi: 10.1136/bmjopen-2017-017631.

The previous version of this manuscript contains an error in using both ICTP-8203 and ICTP-8203Fe as only ICTP-8203Fe. Both designations should read as Dhanashakti.

