# Supplementary material for: Effect of iron and zinc-biofortified pearl millet consumption on growth and immune competence in children aged 12–18 months in India: study protocol for a randomised controlled trial
Source: BMJ Open. 2017 Nov 14;7(11):e017631. doi: 10.1136/bmjopen-2017-017631 (PMC5695508; doi:10.1136/bmjopen-2017-017631)
Supplement: Author's manuscript [file bmjopen-2017-017631.draft_revisions.pdf]

BMJ Open is committed to open peer review. As part of this commitment we make the peer review history of every article we publish publicly available.

When an article is published we post the peer reviewers' comments and the authors' responses online. We also post the versions of the paper that were used during peer review. These are the versions that the peer review comments apply to.

The versions of the paper that follow are the versions that were submitted during the peer review process. They are not the versions of record or the final published versions. They should not be cited or distributed as the published version of this manuscript.

BMJ Open is an open access journal and the full, final, typeset and author-corrected version of record of the manuscript is available on our site with no access controls, subscription charges or pay-per-view fees (http://bmjopen.bmj.com).

If you have any questions on BMJ Open's open peer review process please email editorial.bmjopen@bmj.com

BMJ Open

Effect of Iron- and Zinc-Biofortified Pearl Millet Consumption on Growth, Immune Competence and Cognitive Function in Children Aged 12-18 Months in India—Study Protocol for a Randomised Controlled Trial

Journal:	*BMJ Open*
Manuscript ID	bmjopen-2017-017631
Article Type:	Protocol
Date Submitted by the Author:	03-May-2017
Complete List of Authors:	Mehta, Saurabh; Cornell University ; Institute for Nutritional Sciences, Global Health, and Technology<br>Finkelstein, Julia; Cornell University , Division of Nutritional Sciences; St John's Research Institute<br>Venkatramanan, Sudha; Cornell University , Division of Nutritional Sciences<br>Huey, Samantha; Cornell University , Division of Nutritional Sciences<br>Udipi, Shobha; Kasturba Health Society<br>Ghugre, Padmini; SNDT Women's University, Food Science and Nutrition<br>Ruth, Caleb; Data Performance LLC<br>Canfield, Richard; Cornell University , Division of Nutritional Sciences<br>Kurpad, Anura; St John's Research Institute, Nutrition<br>Potdar, Ramesh; Centre for the Study of Social Change<br>Haas, Jere; Cornell University , Division of Nutritional Sciences
**Primary Subject Heading**:	Global health
Secondary Subject Heading:	Nutrition and metabolism, Paediatrics, Public health
Keywords:	Biofortification, zinc, iron, pearl millet, children, growth

SCHOLARONE™ Manuscripts

Effect of Iron- and Zinc-Biofortified Pearl Millet Consumption on Growth, Immune Competence and Cognitive Function in Children Aged 12-18 Months in India—Study Protocol for a Randomised Controlled Trial

Saurabh Mehta[1,2,3], Julia L. Finkelstein[1,2,3], Sudha Venkatramanan[1], Samantha L. Huey[1], Shobha A. Udipi[4], Padmini Ghugre[5], Caleb Ruth [6], Richard L. Canfield[1], Anura V. Kurpad[3], Ramesh D. Potdar[7], Jere D. Haas[1]

[1]Division of Nutritional Sciences, Cornell University, Ithaca, New York, US

[2]Institute for Nutritional Sciences, Global Health, and Technology, Cornell University, Ithaca, New York, US

[3]St. John's Research Institute, Bangalore, India (SJRI)

[4]Kasturba Health Society Medical Research Centre (KHS-MRC), Mumbai, India

[5]Shreemati Nathibai Damodar Thackersey, Women's University (SNDT), Mumbai, India

[6] Data Performance LLC, New York City, New York, US

[7]Center for the Study of Social Change, Mumbai, India (CSSC)

Corresponding Author (Principal Investigator):

Saurabh Mehta, MBBS, ScD

314 Savage Hall, Ithaca, New York 14853, US

Phone: +1-607-255-2640; Fax: +1-607-255-1033

E-mail: smehta@cornell.edu

Tables and Figures: 1

For peer review only

Keywords: Biofortification, iron, zinc, pearl millet, children, growth

ABSTRACT

Introduction: Biofortified crops represent a sustainable agricultural solution for the widespread micronutrient malnutrition in India and other resource-limited settings. This study aims to investigate the effect of the consumption of iron and zinc-biofortified pearl millet by children on biomarkers of iron and zinc status and growth outcomes. Additionally, we will assess immune and cognitive function in the participants.

Methods and Analysis: We will conduct a randomised controlled feeding trial in identified slums of Mumbai, India among 250 children between 12-18 months of age for 9 months. Children will be randomised to receive the biofortified pearl millet (FeZn-PM, ICTP8203-Fe) or non-biofortified pearl millet. Anthropometric and morbidity data will be gathered every month for 9 months. Biological samples will be collected at baseline, midline, and endline, to assess iron and zinc status, including haemoglobin, serum ferritin, serum transferrin receptor, serum zinc, C-reactive protein, and alpha-1 acid glycoprotein, and other biomarkers. The midline measurement will be a random serial sample between baseline and endline. Immune function and cognitive function will be assessed at each time point by the measurement of vaccine responses and *in-vitro* functional assays in a subset and cognition tests, respectively.

Ethics and Dissemination: This study has obtained clearance from the Health Ministry Screening Committee (HMSC) of the Indian Council of Medical Research (ICMR). Ethical clearance has been obtained from Cornell University's Institutional

For peer review only

Review Board, the Inter System Biomedica Ethics Committee (ISBEC), and St. John's Research Institute Institutional Ethics Review Board. The results of this study will be disseminated at several research conferences and as published articles in peer-reviewed journals.

Registration Details: Clinicaltrials.gov registration number: NCT02233764 (registered on September 4, 2014). Clinical Trials Registry of India (CTRI), reference number REF/2014/10/007731, CTRI number CTRI/2015/11/006376.

For peer review only

Strengths and limitations of this study

- This is the first longitudinal randomised controlled trial to determine the efficacy of consuming complementary foods prepared using iron-biofortified pearl millet on both nutritional status and functional outcomes, among children 12-18 months of age.
- The longitudinal random midline serial sampling strategy both increases sensitivity and the power of the proposed study, while reducing cost and invasiveness (by decreasing the number of biological samples from each participant).
- Data will be collected on iPads or laptops, using a mobile electronic data capture system framework on the iOS platform. This will decrease the potential for error in data entry compared to standard written hard copies of forms, and allow direct uploading of data using a secure server.
- One limitation of this study is that blinding may not be possible due to potential sensory differences between the crops used in the two arms of the study.

For peer review only

INTRODUCTION

The burden of iron and zinc deficiency

Deficiencies in iron and zinc are widely seen as two of the most important public health problems globally[1 2]. The impacts of iron and zinc deficiency on growth[3 4], immune competence[5], and cognitive function[1 6] affect quality of life, particularly in early childhood. The role of iron deficiency in growth and development of children has been demonstrated in several studies[3 7 8]. Similarly, it appears that a major fraction of the growth retardation or stunting can be explained by zinc deficiency [4]. Suboptimal iron and zinc status impairs immune functioning through a number of mechanisms, including a reduction in the proportion of circulating T-lymphocytes and lymphocyte proliferative responses[9]. Cognitive deficits are also observed with iron and zinc deficiency, including irreversible impairments in neurological and psychomotor development of children[10].

Iron and zinc biofortification

Biofortification has the potential to be a more sustainable and cost-effective approach compared to other strategies such as diet diversification, fortification, and supplementation, to address micronutrient malnutrition among vulnerable populations[11]. Previous research from southern India and preliminary data from our acceptability study indicate that the mean consumption of pearl millet (PM) flour in children is 61-80 g/day[12 13]. The iron and zinc content in biofortified PM is reported to be 70-85 and 35-40 parts per million (ppm), respectively. Thus, for children, iron and zinc intakes from PM would be 7-8 mg/100g and 3-4 mg/100g, respectively, and would help meet 50 to 70% of the recommended dietary allowances (RDA) for children between 1-3 years of age. These estimates are based on the RDA recently

For peer review only

recommended for Indians by the Indian Council of Medical Research[14].

The primary objectives of this randomised controlled trial are to study the effect of the daily consumption of high iron- and zinc-biofortified pearl millet on biomarkers of iron and zinc status, as defined by haemoglobin, serum ferritin, serum transferrin receptor, serum zinc, and C-reactive protein; and growth, as defined by length-for-age, weight-for-age and weight-for-length, among 12-18-month-old children, as compared to children receiving conventional pearl millet. Additionally, we will assess the effect of high iron- and zinc-biofortified pearl millet by 12-18-month-old children on immune and cognitive outcomes.

METHODS AND ANALYSIS

Study setting

This study will take place in identified slums of urban Mumbai, Maharashtra, India. Mumbai is the commercial capital of India, and about 41.3% of city's populace resides in urban slums[15].

Study design

We will conduct a randomised controlled trial in which children (12 to 18 months old) will be fed complementary foods prepared using iron- and zinc-biofortified pearl millet (FeZn-PM) or the comparator conventional pearl millet for nine months. Depending on the number of children in the appropriate age group, slums will be selected for the intervention. The participants will be children between the ages of 12 and 18 months at enrolment and will be selected based on the following selection process:

For peer review only

1. Census: Before the study begins, a census will be conducted in the identified slums to gather information on the age of the child, sex, and location.

2. Screening Phase 1: Non-invasive data will be collected on: dietary allergies, use of iron or zinc dietary supplements, availability of a caregiver, and if the caregivers are planning to stay in the slum for the duration of the study. If the children were not dewormed recently, they will be dewormed using liquid albendazole at the study clinic.

3. Screening Phase 2: Blood will be collected during this phase of screening to measure complete blood counts including haemoglobin. Anthropometry and dietary intake will also be measured at this phase of screening.

4. Randomization: The Cornell Statistical Consulting Unit will generate the random allocation sequence using a statistical software package; the randomization sequence key will not be available to any study personnel until follow-up is over. Randomization will be allocated at the individual level. Individuals will be randomized in blocks of 60. Pearl millet food products will coded by study arm (Arm 1: iron- and zinc-biofortified pearl millet; Arm 2: conventional pearl millet). Children will be randomized to either study arm and will be monitored to ascertain they consume the assigned food product throughout the study duration. The midline measurement (including biological sample collection and cognitive assessment) will be a random serial sample taken at any month (months 2-7) between the baseline and endline measurements[16]. Longitudinal midline random serial sampling is often used in population pharmacokinetic research and has been shown to be a useful strategy for

For peer review only

iron fortification efficacy studies, by describing the pattern of iron repletion[16].

Intervention and comparator

The intervention is iron-and-zinc-biofortified pearl millet (ICTP-8203) developed by the International Crops Research Institute for the Semi-Arid Tropics (ICRISAT), and the comparator is a conventional pearl millet that is commercially available on the market. The comparator was chosen because it is similar to the intervention in all aspects, except for iron and zinc content, which allows direct analysis of the impact of the iron-and-zinc-biofortified variety on our outcome measurements. We expect a child to consume an average of 60 g of pearl millet per day, depending on the age of the child[12].

Inclusion and exclusion criteria

Inclusion criteria

Participants included in this study will be 12-18-month-old male and female children with haemoglobin concentration greater than 9 g/dL, living in urban slums of Mumbai.

Exclusion criteria

Children will be excluded if: (1) they are younger than 12 months old or older than 18 months at enrolment; (2) their haemoglobin concentration is less than 9 g/dL or haemoglobinopathy is present (as indicated via abnormal peak via hemoglobin variant analysis); (3) they show signs of severe malnutrition (a weight-for-length, length-for-age, and/or weight-for-age z-score less than -3)[17]; (4) their caregiver reports known diagnosis of diseases such as HIV/AIDS, malaria, tuberculosis, and/or

For peer review only

dengue fever; (5) their caregiver is unavailable to bring the child to the feeding centre during follow-up; (6) the child has any known dietary allergies; and (7) their caregivers are not planning to stay in the study area over the period of follow-up; (8) prior (within the past one year) or current consumption of iron or zinc supplements. Children who are severely anemic will be referred to physicians at the Center for the Study of Social Change (CSSC).

For peer review only

Informed consent process

Research Assistants will obtain informed consent from caregivers in the following stages:

1) *Screening Phase 1*: Based on the census data, informed consent for the collection of data regarding child’s date of birth, deworming, morbidities, and dietary history will be collected. Based on the child’s eligibility at this stage, the child’s caregiver will then be requested to return for the collection of biological specimens.
2) *Screening Phase 2:* Informed consent will be collected from the caregivers for collection and storage of biological samples, as well as anthropometry screening. Blood will be collected to assess haemoglobin concentration. Anthropometric measurements and dietary intake will be collected from caregivers and their children to determine maternal and participant nutritional status and nutrient intake, respectively.
3) *Intervention*: If a child is deemed eligible, the caregiver will be requested to provide informed consent for the child’s enrolment into the trial including consent to storage of biological samples. All trial details will be explained orally and in the written local language (Hindi) by study staff, and each

informed consent process will be recorded by video as per the requirement of the Government of India. Our anticipated date of enrolment of the first participant is June 1, 2017.

Feeding Centre

Feeding centres will be located near to where children and their caregivers' dwellings are clustered, to ensure that travel time from their home to the feeding centre is within walking distance.

Sample size estimation

Our estimates of sample size are based on assumptions about mean values and associated variation in haemoglobin and serum ferritin. We expect 50–75% of these children to have iron and zinc deficiency based on published literature[18] and preliminary results (unpublished). We assume that absorbed iron will be transferred to body stores in the liver, and that the change in liver stores is reflected in changes in serum ferritin at a rate of 8 μg/L of serum ferritin per mg liver iron in non-anaemic, iron-depleted children. Iron-deficient, anaemic (haemoglobin < 11 g/dL[19]) children will have their absorbable iron directed to haemoglobin synthesis in the early months of the feeding trial. We estimate that the children consuming biofortified FeZn-PM will demonstrate a gain of 1 g/dL in haemoglobin concentrations in 2.4 months, whereas children consuming control pearl millet will need more than 9 months to demonstrate the same increase. To detect a significant increase in ferritin from the baseline value of 1.79 (6 mg/L) with standard deviation of 1.2, in the experimental group, compared to the control group, at a power of 80% and 5% significance level, 96 participants will be required. We propose a larger sample size of n=250, to

For peer review only

ensure adequate power for other outcomes of interest in this study, including immune and cognitive function, as well as biomarkers for zinc status which are less defined than biomarkers of iron status.

Follow-up

Assessments

At baseline, we will assess anthropometry; collect blood for analysis of hemoglobin, iron, zinc, CRP, and AGP biomarkers in the blood (all blood analyses other than complete blood counts will be performed in batch at the end of the trial to minimize error); nutrient intake measures using multiple-pass 24-h dietary recall[20] for children and food frequency questionnaire for the mothers; socio-economic and demographic information; and morbidity history. Follow-up will continue for 9 months and will include monthly anthropometric measurements, morbidity assessments, and 24-hour dietary recall. Additionally, we will conduct cognitive testing and collect blood and stool samples to measure immune function at three time points (baseline, midline and endline). The midline time point will be a random serial sample that occurs between the baseline and endline measures. Children may visit the CSSC clinic at any time during the trial for healthcare treatment.

Administration of intervention

Both arms will receive complementary foods prepared with pearl millet three times per day, 6 days per week, for 9 months. Each day, the child's caregiver will bring the child to their feeding centre. Two meals will be consumed at the feeding centre; the third meal may be consumed at home. The food intake of the two meals at the feeding centre will be measured directly before and after consumption. To measure

For peer review only - http://bmjopen.bmj.com/site/about/guidelines.xhtml

the intake of the third meal, the weight of unconsumed food from the third meal will be measured the next morning at the feeding centre. To assure adherence, healthcare workers will follow up with the participants' caregivers and record reasons for non-adherence. Throughout the study, we will conduct periodic analysis of random samples of both grain varieties and prepared food products to ensure food safety and quality of the intervention.

Primary outcome measurements

Biomarkers of iron and zinc status

We will determine if biofortified pearl millet improves iron and zinc status compared to children who consume non-biofortified pearl millet. Specifically, iron and zinc status will be assessed by measuring concentrations of haemoglobin (Hb), serum ferritin, serum transferrin receptor (sTfR) and plasma zinc at enrolment (baseline), midline, and endline. Additionally, we will measure concentrations of inflammatory biomarkers C-reactive protein (CRP) and alpha 1-acid glycoprotein (AGP), as iron and zinc biomarkers can be influenced by inflammation.

Growth

We will assess change in a child's growth as another outcome variable to determine if iron-biofortified pearl millet reduces the risk of underweight (weight-for-age z score < -2), wasting (weight-for-height z-score < -2) and stunting (length-for-age z score < -2) during the 9-month intervention period, compared to those who receive non-biofortified pearl millet. We will measure weight (kg) and length (cm) at baseline and monthly throughout follow-up. The weight and length data will be converted to weight-for-age, weight-for-length z-score, and length-for-age z-score. The growth

For peer review only

rates (as kg/month, and cm/month) will also be compared from the absolute measurements after controlling for age.

Additional outcomes

Immune function

We will directly assess immune function by measuring superoxide burst and proteolytic capacities of macrophages and monocytes. Activity index will be assessed by comparing mean substrate and calibration florescence[21]. Additionally, we will collect morbidity data, including changes in types and frequencies of morbidities such as diarrhoeal illness, pneumonia, and any chronic disease throughout follow-up during each clinic visit. Caregivers will also be encouraged to come to the clinic at any time for any health-related reasons, which will also be recorded. This will provide an accurate representation of both innate and adaptive immunity in participants.

Cognitive function

To determine if consumption of biofortified pearl millet improves child cognitive function, compared to consumption of non-biofortified pearl millet, we will assess aspects of cognition that (1) previously have been shown to be sensitive to the effects of early iron and/or zinc deficiency, or (2) draw heavily upon brain structures or processes thought to be vulnerable to early iron and/or zinc deficiency, based on studies of animal models and humans. Thus, we will assess multiple specific aspects of memory, attention and processing speed using automated eye trackers[22]. In addition, we will also assess higher-level, integrative cognitive abilities, including problem-solving and exploratory behavior and global aspects of attention and

For peer review only

inattention during free play with toys[23]. Finally, we will conduct a Developmental Assessment Scales for Indian Infants (DASII)[24] test at the baseline, intermediate and endline assessments to obtain a broad measure of attainment of developmental milestones that can be used to compare the characteristics of our cohort with those reported in the literature.

Data collection and storage

Prior to data collection, the field staff will be trained on ethical and data collection procedures. The data collection tools will be translated from English into the local languages of Hindi and back-translated into English to retain the original meaning. The Hindi regional language versions of the questionnaire will be used for data collection. A pre-testing of the data collection tools will be evaluated before the main intervention; the protocol of data collection forms to be used is shown in Figure 1. The data collection will begin with baseline measurements in January 2017. All data will be collected on iPads or laptops for the proposed project. We will use a mobile electronic data capture (mEDC) framework on the iOS platform, *Connedct,* that was created by our Database Developer and is being used in our ongoing efficacy trial in Mumbai [25]. A secure server will be utilized for uploading, storing, and accessing the data. This will enable real-time feedback and error checking, as well as eliminate errors associated with data entry, facilitating faster data analysis and rapid dissemination of results. All information will be kept confidential. All names will be removed from the data for analysis. The identifying codes and linked names will be securely stored in password-protected computers. All data will be securely stored on a third-party server, which will have limited access by study team members. All biological specimens will be collected by trained medical professionals and

For peer review only

evaluated by certified laboratories within India. Biological specimens will be stored appropriately throughout the duration of the study and after the study for future analysis. All study investigators will have access to the final trial dataset via contractual agreements.

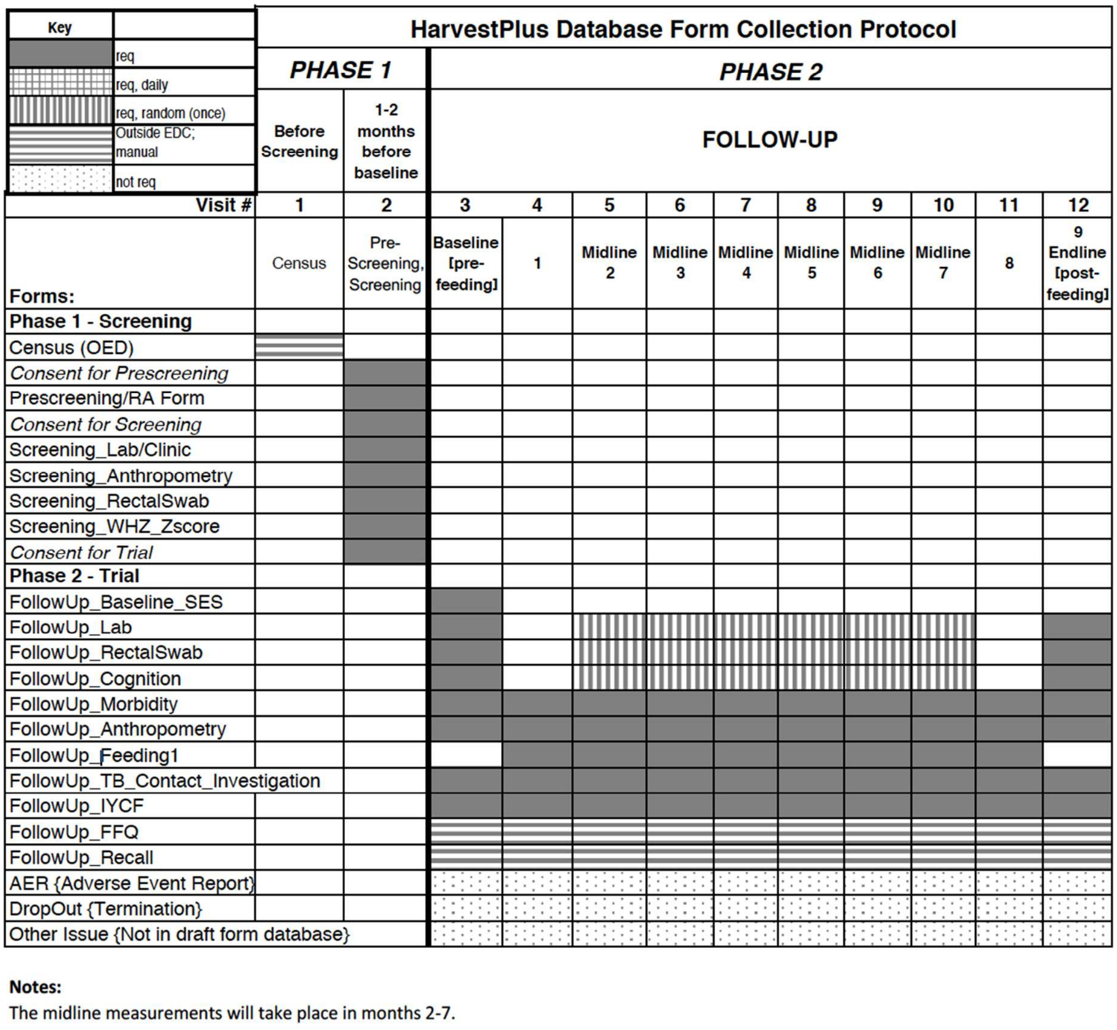

Figure 1. Form Collection Protocol.

Data analysis

We will use an intention-to-treat approach to determine the effect of biofortified pearl millet on the outcomes described above. Advanced analysis will use mixed models to account for pearl millet-based complementary foods consumed and use each

block of 60 as a random effect. We will also plot dose-response curves using restricted cubic splines that will help us detect any threshold effects, as well as non-linear associations. Nonparametric tests, such as the Hodges-Lehmann test, will be used where relevant, as in the case of non-normally distributed variables including serum ferritin.

Study monitoring board

A Data Safety Monitoring Board (DSMB) will be established for this study and will include experts representing Cornell University and SNDT University who are not directly involved in the trial. The DSMB members will oversee the study and will periodically monitor the progress and outcomes of the intervention.

Reporting of adverse events

All significant adverse events will be reported by the study physican via an Adverse Event/Serious Adverse Event Report (AE/SAE) form. When an adverse event occurs, study physicians, research assistants and/or other study personnel will undertake all necessary precautions to ensure the safety and well-being of the participant. Participants will not receive any compensation or payment for taking part in the study. Every participant will be insured for the duration of the trial in the unexpected and unlikely event of any adverse event relating to the study.

The following study protocol endpoints will be considered to define safety and efficacy outcomes, and establish un-blinding and stopping guidelines in this trial, as per the discretion of the DSMB: 1. Diagnosis of the development of severe acute malnutrition such as Kwashiorkor; 2. Occurrence of all-cause death. Demonstration of efficacy, namely a significant beneficial effect on mortality and other adverse

For peer review only

outcomes will be used as a guideline to determine if the study should be un-blinded, stopped, or terminated.

ETHICS AND DISSEMINATION

Before conducting interviews or allowing participation in the study, written informed consent will be obtained from each participant's guardians/primary caregivers, and research assistants will record each granting of informed consent using audio and video techniques. We will also assess children's current nutritional status to ensure that our study provision is at least comparable to the children's daily dietary intake, which is generally lower than ideal for growth and health, according to preliminary data. Severely anaemic (<7 g/dL) and malnourished children will be provided with appropriate medical care and referred to CSSC. The caregivers will be assured of the confidentiality and anonymity of reports and publications generated from this study. Participation will be voluntary, and participants will be assured that refusal to participate in the study will not impact their access to care. The ethical clearance for this study has been obtained from the Institutional Review Board of Cornell University; Intersystem Biomedical Ethics Committee (ISBEC) of SNDT University, Mumbai, India; and the Institutional Review Board of St. John's Research Institute, Bangalore, India. This study has received clearance from Health Ministry's Screening Committee (HMSC) of the ICMR. The results of this study will be disseminated at several research conferences and as published articles in peer-reviewed journals. The present study protocol was prepared in accordance to the SPIRIT statement[26]. This trial has been registered at Clinicaltrials.gov (registration number NCT02233764) and the Clinical Trials Registry of India (CTRI) (reference

For peer review only

number REF/2014/10/007731, CTRI number CTRI/2015/11/006376).

REFERENCES

1. Black MM, Quigg AM, Hurley KM, et al. Iron deficiency and iron-deficiency anemia in the first two years of life: strategies to prevent loss of developmental potential. *Nutrition reviews* 2011;69 Suppl 1:S64-70. doi: 10.1111/j.1753-4887.2011.00435.x
2. Black RE, Victora CG, Walker SP, et al. Maternal and child undernutrition and overweight in low-income and middle-income countries. *The Lancet* 2013;382(9890):427-51. doi: 10.1016/s0140-6736(13)60937-x
3. Vucic V, Berti C, Vollhardt C, et al. Effect of iron intervention on growth during gestation, infancy, childhood, and adolescence: a systematic review with meta-analysis. *Nutrition reviews* 2013;71(6):386-401. doi: 10.1111/nure.12037
4. Cole CR, Lifshitz F. Zinc nutrition and growth retardation. *Pediatr Endocrinol Rev* 2008;5(4):889-96.
5. Oppenheimer SJ. Iron and its relation to immunity and infectious disease. *The Journal of nutrition* 2001;131(2S-2):616S-33S; discussion 33S-35S.
6. Black MM. The evidence linking zinc deficiency with children's cognitive and motor functioning. *The Journal of nutrition* 2003;133(5 Suppl 1):1473S-6S.
7. Lawless J, Latham M, Kinoti S, et al. Iron Supplementation Improves Appetite and Growth in Anemic Kenyan Primary School Children. *Journal of Nutrition* 1994;124:645-54.
8. Kanani S, RH P. Symposium Supplementation with Iron and Folic Acid Enhances Growth in Adolescent Indian Girls. *Journal of Nutrition* 2000;130(452S-455S)
9. Walter T, Olivares M, Pizarro F, et al. Iron, anemia, and infection. *Nutrition reviews* 1997;55(4):111-24.
10. Georgieff MK. Long-term brain and behavioral consequences of early iron deficiency. *Nutrition reviews* 2011;69 Suppl 1:S43-8. doi: 10.1111/j.1753-4887.2011.00432.x
11. HE B. Plant Breeding- A New Tool for Fighting Micronutrient Malnutrition. *Am Soc Nutr Sci* 2002:511-3.
12. Acceptability of iron biofortified pearl millet among young children in urban slums of Mumbai, India. Experimental Biology; 2016; San Diego. FASEB journal : official publication of the Federation of American Societies for Experimental Biology.
13. Kodkany BS, Bellad RM, Mahantshetti NS, et al. Biofortification of pearl millet with iron and zinc in a randomized controlled trial increases absorption of these minerals above physiologic requirements in young children. *The Journal of nutrition* 2013;143(9):1489-93. doi: 10.3945/jn.113.176677
14. Research EGotICoM. Nutrient Requirements and Recommended Dietary Allowances for Indians. In: Research EGotICoM, ed. Hyderabad, India: National Institute of Nutrition, Indian Council of Medical Research, 2009.

For peer review only

15. Chandramouli C. HOUSING STOCK, AMENITIES & ASSETS IN SLUMS - CENSUS 2011 (Presentation). In: Affairs MoH, ed. India: Census of India, 2011.
16. Andersson M, Theis W, Zimmermann MB, et al. Random serial sampling to evaluate efficacy of iron fortification: a randomized controlled trial of margarine fortification with ferric pyrophosphate or sodium iron edetate. *The American journal of clinical nutrition* 2010;92(5):1094-104. doi: 10.3945/ajcn.2010.29523
17. de Onis M, Onyango A, Borghi E, et al. Worldwide implementation of the WHO Child Growth Standards. *Public health nutrition* 2012;15(9):1603-10. doi: 10.1017/S136898001200105X
18. Kapoor D, Agarwal KN, Sharma S, et al. Iron status of children aged 9-36 months in an urban slum Integrated Child Development Services project in Delhi. *Indian Pediatr* 2002;39(2):136-44.
19. WHO/NMH/NHD/11.1. Haemoglobin concentrations for the diagnosis of anaemia and assessment of severity. 2011
20. Rizek RL, Pao EM. Dietary intake methodology I. USDA surveys and supporting research. *The Journal of nutrition* 1990;120 Suppl 11:1525-9.
21. Podinovskaia M, VanderVen BC, Yates RM, et al. Dynamic quantitative assays of phagosomal function. *Curr Protoc Immunol* 2013;102:Unit 14 34. doi: 10.1002/0471142735.im1434s102
22. Aslin R, B M. Automated Corneal-Reflection Eye Tracking in Infancy- Methodological Developments and Applications to Cognition. *Infancy* 2004;6:155-63. doi: 10.1207/s15327078in0602_1
23. Ruff HA, Lawson KR, Parrinello R, et al. Long-term stability of individual differences in sustained attention in the early years. *Child Dev* 1990;61(1):60-75.
24. Phatak P. Developmental Assessment Scales for Indian Infants (DASII)- Revised Baroda Norms Manual. *Pune: Anand Agencies* 1998
25. Ruth C, Yu EA, Huey SL, et al. ConnEDCt: A Development Framework for Mobile Electronic Data Capture in Disconnected Communities. *Computer Science and Education in Computer Science* 2016;12:219-33.
26. Chan A-W, Tetzlaff J, Altman D, et al. SPIRIT 2013 Statement- Defining Standard Protocol Items for Clinical Trials. *Ann Intern Med* 2013;158:200-7.

AUTHORS' CONTRIBUTIONS: Saurabh Mehta, Julia L. Finkelstein and Jere D. Haas designed the study, conceived the research questions and prepared the study protocol. Saurabh Mehta is the principal investigator of the study and Julia L. Finkelstein, Jere D. Haas, Shobha A. Udipi, Padmini Ghugre, Richard L. Canfield, Anura V. Kurpad, and Ramesh D. Potdar and are co-investigators. Caleb Ruth developed the database used in this trial and provided feedback on the protocol. Samantha L. Huey and Sudha Venkatramanan contributed to the editing of the

For peer review only

protocol and will supervise the data collection under the guidance of the investigators. All authors read and approved the final protocol.

ABBREVIATIONS: AGP: Alpha 1-acid glycoprotein; CRP: C-reactive protein; Fe: Iron; FeZn-PM: iron- and zinc-enhanced variety of pearl millet; ICRISAT: International Crops Research Institute for the Semi-Arid Tropics; ICTP8203-Fe: high iron and zinc-biofortified pearl millet variety; ICMR: Indian Council of Medical Research; ISBEC: Intersystem Biomedical Ethics Committee; IRB: Institutional Review Board; KHS: Kasturba Health Society; PM: Pearl Millet; RDA: Recommended Daily Allowance; SJRI: St. John's Research Institute; SNDT: Shreemati Nathibai Damodar Thackersey; sTfR: Serum transferrin receptor; Zn: Zinc

FUNDING: This work was supported by HarvestPlus, grant number 2014H8302.

Address:

HarvestPlus

2033 K St NW

Washington, DC 20006

CONFLICTS OF INTEREST: SM is an unpaid board member for a diagnostic start up focused on developing point-of-care assays for nutritional status informed by his research as a faculty member at Cornell University. All other authors report no conflict of interest.

For peer review only

Key	
(dark grey)	req
(grid)	req, daily
(vertical stripes)	req, random (once)
(horizontal stripes)	Outside EDC; manual
(dotted)	not req

HarvestPlus Database Form Collection Protocol

	PHASE 1		*PHASE 2*									
	Before Screening	1-2 months before baseline	FOLLOW-UP									
Visit #	1	2	3	4	5	6	7	8	9	10	11	12
Forms:	Census	Pre-Screening, Screening	Baseline [pre-feeding]	1	Midline 2	Midline 3	Midline 4	Midline 5	Midline 6	Midline 7	8	9 Endline [post-feeding]
**Phase 1 - Screening**												
Census (OED)	Outside EDC; manual											
Consent for Prescreening		req										
Prescreening/RA Form		req										
Consent for Screening		req										
Screening_Lab/Clinic		req										
Screening_Anthropometry		req										
Screening_RectalSwab		req										
Screening_WHZ_Zscore		req										
Consent for Trial		req										
**Phase 2 - Trial**												
FollowUp_Baseline_SES			req									
FollowUp_Lab			req		req, random (once)	req, random (once)	req, random (once)	req, random (once)	req, random (once)	req, random (once)		req
FollowUp_RectalSwab			req		req, random (once)	req, random (once)	req, random (once)	req, random (once)	req, random (once)	req, random (once)		req
FollowUp_Cognition			req		req, random (once)	req, random (once)	req, random (once)	req, random (once)	req, random (once)	req, random (once)		req
FollowUp_Morbidity			req	req	req	req	req	req	req	req	req	req
FollowUp_Anthropometry			req	req	req	req	req	req	req	req	req	req
FollowUp_Feeding1				req	req	req	req	req	req	req	req	
FollowUp_TB_Contact_Investigation			req	req	req	req	req	req	req	req	req	req
FollowUp_IYCF			req	req	req	req	req	req	req	req	req	req
FollowUp_FFQ			Outside EDC; manual	Outside EDC; manual	Outside EDC; manual	Outside EDC; manual	Outside EDC; manual	Outside EDC; manual	Outside EDC; manual	Outside EDC; manual	Outside EDC; manual	Outside EDC; manual
FollowUp_Recall			Outside EDC; manual	Outside EDC; manual	Outside EDC; manual	Outside EDC; manual	Outside EDC; manual	Outside EDC; manual	Outside EDC; manual	Outside EDC; manual	Outside EDC; manual	Outside EDC; manual
AER {Adverse Event Report}			not req	not req	not req	not req	not req	not req	not req	not req	not req	not req
DropOut {Termination}			not req	not req	not req	not req	not req	not req	not req	not req	not req	not req
Other Issue {Not in draft form database}			not req	not req	not req	not req	not req	not req	not req	not req	not req	not req

Notes:
The midline measurements will take place in months 2-7.

Figure 1. Form Collection Protocol.

only

SPIRIT
STANDARD PROTOCOL ITEMS: RECOMMENDATIONS FOR INTERVENTIONAL TRIALS

SPIRIT 2013 Checklist: Recommended items to address in a clinical trial protocol and related documents*

Section/item	Item No	Description	Addressed on page number
**Administrative information**			
Title	1	Descriptive title identifying the study design, population, interventions, and, if applicable, trial acronym	_1____________
Trial registration	2a	Trial identifier and registry name. If not yet registered, name of intended registry	_3,16__________
	2b	All items from the World Health Organization Trial Registration Data Set	_yes_________
Protocol version	3	Date and version identifier	_N/A________
Funding	4	Sources and types of financial, material, and other support	_19
Roles and responsibilities	5a	Names, affiliations, and roles of protocol contributors	_1, 18________
	5b	Name and contact information for the trial sponsor	_19____________
	5c	Role of study sponsor and funders, if any, in study design; collection, management, analysis, and interpretation of data; writing of the report; and the decision to submit the report for publication, including whether they will have ultimate authority over any of these activities	_18____________
	5d	Composition, roles, and responsibilities of the coordinating centre, steering committee, endpoint adjudication committee, data management team, and other individuals or groups overseeing the trial, if applicable (see Item 21a for data monitoring committee)	_15____________

For peer review only - http://bmjopen.bmj.com/site/about/guidelines.xhtml

**Introduction**			
Background and rationale	6a	Description of research question and justification for undertaking the trial, including summary of relevant studies (published and unpublished) examining benefits and harms for each intervention	_5, 6__________
	6b	Explanation for choice of comparators	_7, 8____________
Objectives	7	Specific objectives or hypotheses	_6_____________
Trial design	8	Description of trial design including type of trial (eg, parallel group, crossover, factorial, single group), allocation ratio, and framework (eg, superiority, equivalence, noninferiority, exploratory)	_6____________
**Methods: Participants, interventions, and outcomes**			
Study setting	9	Description of study settings (eg, community clinic, academic hospital) and list of countries where data will be collected. Reference to where list of study sites can be obtained	_6_____________
Eligibility criteria	10	Inclusion and exclusion criteria for participants. If applicable, eligibility criteria for study centres and individuals who will perform the interventions (eg, surgeons, psychotherapists)	__8,9______
Interventions	11a	Interventions for each group with sufficient detail to allow replication, including how and when they will be administered	_9,10___________
	11b	Criteria for discontinuing or modifying allocated interventions for a given trial participant (eg, drug dose change in response to harms, participant request, or improving/worsening disease)	_11_____________
	11c	Strategies to improve adherence to intervention protocols, and any procedures for monitoring adherence (eg, drug tablet return, laboratory tests)	_12_____________
	11d	Relevant concomitant care and interventions that are permitted or prohibited during the trial	_10,11__________
Outcomes	12	Primary, secondary, and other outcomes, including the specific measurement variable (eg, systolic blood pressure), analysis metric (eg, change from baseline, final value, time to event), method of aggregation (eg, median, proportion), and time point for each outcome. Explanation of the clinical relevance of chosen efficacy and harm outcomes is strongly recommended	_11-13_________
Participant timeline	13	Time schedule of enrolment, interventions (including any run-ins and washouts), assessments, and visits for participants. A schematic diagram is highly recommended (see Figure)	_x (see attached figure) __

For peer review only - http://bmjopen.bmj.com/site/about/guidelines.xhtml

Sample size	14	Estimated number of participants needed to achieve study objectives and how it was determined, including clinical and statistical assumptions supporting any sample size calculations	__10,11_______
Recruitment	15	Strategies for achieving adequate participant enrolment to reach target sample size	_10,11______

Methods: Assignment of interventions (for controlled trials)

Allocation:

Sequence generation	16a	Method of generating the allocation sequence (eg, computer-generated random numbers), and list of any factors for stratification. To reduce predictability of a random sequence, details of any planned restriction (eg, blocking) should be provided in a separate document that is unavailable to those who enrol participants or assign interventions	_7________
Allocation concealment mechanism	16b	Mechanism of implementing the allocation sequence (eg, central telephone; sequentially numbered, opaque, sealed envelopes), describing any steps to conceal the sequence until interventions are assigned	_7,8______
Implementation	16c	Who will generate the allocation sequence, who will enrol participants, and who will assign participants to interventions	_7____________
Blinding (masking)	17a	Who will be blinded after assignment to interventions (eg, trial participants, care providers, outcome assessors, data analysts), and how	_4__________
	17b	If blinded, circumstances under which unblinding is permissible, and procedure for revealing a participant's allocated intervention during the trial	_N/A________

Methods: Data collection, management, and analysis

Data collection methods	18a	Plans for assessment and collection of outcome, baseline, and other trial data, including any related processes to promote data quality (eg, duplicate measurements, training of assessors) and a description of study instruments (eg, questionnaires, laboratory tests) along with their reliability and validity, if known. Reference to where data collection forms can be found, if not in the protocol	_12-14________
	18b	Plans to promote participant retention and complete follow-up, including list of any outcome data to be collected for participants who discontinue or deviate from intervention protocols	_12, 16_____

For peer review only - http://bmjopen.bmj.com/site/about/guidelines.xhtml

Data management	19	Plans for data entry, coding, security, and storage, including any related processes to promote data quality (eg, double data entry; range checks for data values). Reference to where details of data management procedures can be found, if not in the protocol	_14____________
Statistical methods	20a	Statistical methods for analysing primary and secondary outcomes. Reference to where other details of the statistical analysis plan can be found, if not in the protocol	_15____________
	20b	Methods for any additional analyses (eg, subgroup and adjusted analyses)	_15____________
	20c	Definition of analysis population relating to protocol non-adherence (eg, as randomised analysis), and any statistical methods to handle missing data (eg, multiple imputation)	_15____________
**Methods: Monitoring**			
Data monitoring	21a	Composition of data monitoring committee (DMC); summary of its role and reporting structure; statement of whether it is independent from the sponsor and competing interests; and reference to where further details about its charter can be found, if not in the protocol. Alternatively, an explanation of why a DMC is not needed	_15_________
	21b	Description of any interim analyses and stopping guidelines, including who will have access to these interim results and make the final decision to terminate the trial	_16________
Harms	22	Plans for collecting, assessing, reporting, and managing solicited and spontaneously reported adverse events and other unintended effects of trial interventions or trial conduct	_15,16________
Auditing	23	Frequency and procedures for auditing trial conduct, if any, and whether the process will be independent from investigators and the sponsor	_15_________
**Ethics and dissemination**			
Research ethics approval	24	Plans for seeking research ethics committee/institutional review board (REC/IRB) approval	_16,17________
Protocol amendments	25	Plans for communicating important protocol modifications (eg, changes to eligibility criteria, outcomes, analyses) to relevant parties (eg, investigators, REC/IRBs, trial participants, trial registries, journals, regulators)	_16,17_______

For peer review only - http://bmjopen.bmj.com/site/about/guidelines.xhtml

Consent or assent	26a	Who will obtain informed consent or assent from potential trial participants or authorised surrogates, and how (see Item 32)	_9,10________
	26b	Additional consent provisions for collection and use of participant data and biological specimens in ancillary studies, if applicable	_9,10 _
Confidentiality	27	How personal information about potential and enrolled participants will be collected, shared, and maintained in order to protect confidentiality before, during, and after the trial	_14,15__
Declaration of interests	28	Financial and other competing interests for principal investigators for the overall trial and each study site	_20____________
Access to data	29	Statement of who will have access to the final trial dataset, and disclosure of contractual agreements that limit such access for investigators	_15____________
Ancillary and post-trial care	30	Provisions, if any, for ancillary and post-trial care, and for compensation to those who suffer harm from trial participation	_16____________
Dissemination policy	31a	Plans for investigators and sponsor to communicate trial results to participants, healthcare professionals, the public, and other relevant groups (eg, via publication, reporting in results databases, or other data sharing arrangements), including any publication restrictions	_16,17_________
	31b	Authorship eligibility guidelines and any intended use of professional writers	_N/A___________
	31c	Plans, if any, for granting public access to the full protocol, participant-level dataset, and statistical code	_17___________
**Appendices**			
Informed consent materials	32	Model consent form and other related documentation given to participants and authorised surrogates	_attached___
Biological specimens	33	Plans for collection, laboratory evaluation, and storage of biological specimens for genetic or molecular analysis in the current trial and for future use in ancillary studies, if applicable	__15______

*It is strongly recommended that this checklist be read in conjunction with the SPIRIT 2013 Explanation & Elaboration for important clarification on the items. Amendments to the protocol should be tracked and dated. The SPIRIT checklist is copyrighted by the SPIRIT Group under the Creative Commons "Attribution-NonCommercial-NoDerivs 3.0 Unported" license.

For peer review only - http://bmjopen.bmj.com/site/about/guidelines.xhtml

BMJ Open

Effect of Iron- and Zinc-Biofortified Pearl Millet Consumption on Growth and Immune Competence in Children Aged 12-18 Months in India—Study Protocol for a Randomised Controlled Trial

Journal:	*BMJ Open*
Manuscript ID	bmjopen-2017-017631.R1
Article Type:	Protocol
Date Submitted by the Author:	07-Sep-2017

Complete List of Authors:	Mehta, Saurabh; Cornell University ; Institute for Nutritional Sciences, Global Health, and Technology<br>Finkelstein, Julia; Cornell University , Division of Nutritional Sciences; St John's Research Institute<br>Venkatramanan, Sudha; Cornell University , Division of Nutritional Sciences<br>Huey, Samantha; Cornell University , Division of Nutritional Sciences<br>Udipi, Shobha; Kasturba Health Society<br>Ghugre, Padmini; SNDT Women's University, Food Science and Nutrition<br>Ruth, Caleb; Data Performance LLC<br>Canfield, Richard; Cornell University , Division of Nutritional Sciences<br>Kurpad, Anura; St John's Research Institute, Nutrition<br>Potdar, Ramesh; Centre for the Study of Social Change<br>Haas, Jere; Cornell University , Division of Nutritional Sciences

**Primary Subject Heading**:	Global health
Secondary Subject Heading:	Nutrition and metabolism, Paediatrics, Public health
Keywords:	Biofortification, zinc, iron, pearl millet, children, growth

SCHOLARONE™ Manuscripts

Effect of Iron- and Zinc-Biofortified Pearl Millet Consumption on Growth and Immune Competence in Children Aged 12-18 Months in India—Study Protocol for a Randomised Controlled Trial

Saurabh Mehta[1,2], Julia L. Finkelstein[1,3], Sudha Venkatramanan[1], Samantha L. Huey[1], Shobha A. Udipi[4], Padmini Ghugre[5], Caleb Ruth [6], Richard L. Canfield[1], Anura V. Kurpad[3], Ramesh D. Potdar[7], Jere D. Haas[1]

[1]Division of Nutritional Sciences, Cornell University, Ithaca, New York, US

[2]Institute for Nutritional Sciences, Global Health, and Technology, Cornell University, Ithaca, New York, US

[3]St. John's Research Institute, Bangalore, India (SJRI)

[4]Kasturba Health Society Medical Research Centre (KHS-MRC), Mumbai, India

[5]Shreemati Nathibai Damodar Thackersey, Women's University (SNDT), Mumbai, India

[6] Data Performance LLC, Ithaca, New York, US

[7]Center for the Study of Social Change, Mumbai, India (CSSC)

Corresponding Author (Principal Investigator):

Saurabh Mehta, MBBS, ScD

314 Savage Hall, Ithaca, New York 14853, US

Phone: +1-607-255-2640; Fax: +1-607-255-1033

E-mail: smehta@cornell.edu

Tables and Figures: 2

For peer review only

Keywords: Biofortification, iron, zinc, pearl millet, children, growth

ABSTRACT

Introduction: Biofortified crops represent a sustainable agricultural solution for the widespread micronutrient malnutrition in India and other resource-limited settings. This study aims to investigate the effect of the consumption of iron and zinc-biofortified pearl millet by children on biomarkers of iron and zinc status and growth outcomes. Additionally, we will assess immune function in the participants.

Methods and Analysis: We will conduct a randomised controlled feeding trial in identified slums of Mumbai, India among 250 children between 12-18 months of age for 9 months. Children will be randomised to receive the biofortified pearl millet (FeZn-PM, ICTP8203-Fe) or non-biofortified pearl millet. Anthropometric and morbidity data will be gathered every month for 9 months. Biological samples will be collected at baseline, midline, and endline, to assess iron and zinc status, including haemoglobin, serum ferritin, serum transferrin receptor, serum zinc, C-reactive protein, and alpha-1 acid glycoprotein. Biological samples will be archived for future analyses. The midline measurement will be a random serial sample between baseline and endline. Immune function will be assessed at each time point by the measurement of T cell counts in a subset, respectively.

Ethics and Dissemination: This study has obtained clearance from the Health Ministry Screening Committee (HMSC) of the Indian Council of Medical Research (ICMR). Ethical clearance has been obtained from Cornell University's Institutional Review Board, the Inter System Biomedica Ethics Committee (ISBEC), and St. John's Research Institute Institutional Ethics Review Board. The results of this study

For peer review only

will be disseminated at several research conferences and as published articles in peer-reviewed journals.

Registration Details: Clinicaltrials.gov registration number: NCT02233764 (registered on September 4, 2014). Clinical Trials Registry of India (CTRI), reference number REF/2014/10/007731, CTRI number CTRI/2015/11/006376.

For peer review only

Strengths and limitations of this study

- This is the first longitudinal randomised controlled trial to determine the efficacy of consuming complementary foods prepared using iron- and zinc-biofortified pearl millet on both nutritional status and functional outcomes, among children 12-18 months of age.
- The longitudinal random midline serial sampling strategy both increases sensitivity and the power of the proposed study, while reducing cost and invasiveness (by decreasing the number of biological samples from each participant).
- Data will be collected on iPads or laptops, using a mobile electronic data capture system framework on the iOS platform. This will decrease the potential for error in data entry compared to standard written hard copies of forms, and allow direct uploading of data using a secure server.
- One limitation of this study is that blinding may not be possible due to potential sensory differences between the crops used in the two arms of the study.

For peer review only

INTRODUCTION

The burden of iron and zinc deficiency

Deficiencies in iron and zinc are two of the most important public health problems globally[1 2]. The role of iron deficiency in growth and development of children has been demonstrated in several studies[3-5]. Similarly, it appears that a major fraction of stunting can be explained by zinc deficiency[6]. Suboptimal iron and zinc status also impairs immune functioning through a number of mechanisms, including a reduction in the proportion of circulating T-lymphocytes and lymphocyte proliferative responses[7]. Cognitive deficits are also observed with iron and zinc deficiency, including irreversible impairments in neurological and psychomotor development of children[8].

Iron and zinc biofortification

Biofortification has the potential to be a more sustainable and cost-effective approach compared to other strategies such as diet diversification, fortification, and supplementation, to address micronutrient malnutrition among vulnerable populations[9]. In India, staple crops such as pearl millet (*Pennisetum glaucum)* are consumed as part of the daily diet, particularly in Maharashtra, Gujarat, Rajasthan, and Karnataka. The iron and zinc concentration in biofortified PM is reported to be 70-85 and 35-40 parts per million (ppm), respectively[10]. Previous research from southern India and preliminary data from our acceptability study indicate that the mean consumption of pearl millet (PM) flour in children is 61-80 g/day among Indian children[11 12], showing the suitability of this crop as a target for biofortification. Thus, for children, iron and zinc intakes from PM would be 7-8 mg/100g and 3-4 mg/100g, respectively[10], and would help meet 50 to 70% of the recommended dietary

For peer review only

allowances (RDA) for children between 1-3 years of age. These estimates are based on the RDA recently recommended for Indians by the Indian Council of Medical Research[13].

The primary objectives of this randomised controlled trial are to study the effect of the daily consumption of high iron- and zinc-biofortified pearl millet on biomarkers of iron and zinc status, as defined by haemoglobin, serum ferritin, serum transferrin receptor, serum zinc, and C-reactive protein; and growth, as defined by length-for-age, weight-for-age and weight-for-length, among 12-18-month-old children, as compared to children receiving conventional pearl millet. Additionally, we will assess the effect of high iron- and zinc-biofortified pearl millet by 12-18-month-old children on immune outcomes.

METHODS AND ANALYSIS

Study setting

This study will take place in identified slums of urban Mumbai, Maharashtra, India. Mumbai is the commercial capital of India, and about 41.3% of city's populace resides in urban slums[14].

Study design

We will conduct a randomised controlled trial in which children (12 to 18 months old) will be fed complementary foods prepared using iron- and zinc-biofortified pearl millet (FeZn-PM) or the comparator conventional pearl millet for nine months. Caregivers will be requested to provide three (3) informed consent forms for 1) census; 2) screening; and 3) enrolment into the trial. Caregivers who provide all

For peer review only - http://bmjopen.bmj.com/site/about/guidelines.xhtml

three consents are considered eligible to be screened. Details of the informed consent process can be found the section “Informed consent process.”

1. Census: Before the study begins, a census will be conducted in the identified slums to gather information on the age of the child, sex, and location.

2. Screening: Consent will be obtained for screening and inclusion in the trial. Non-invasive data will be collected on: dietary allergies, use of iron or zinc dietary supplements, availability of a caregiver, and if the caregivers are planning to stay in the slum for the duration of the study. Anthropometry and dietary intake will also be measured. If the children were not dewormed recently, they will be provided liquid albendazole by the study physician at the study clinic (5 mL of syrup equivalent to 200 mg per dose). If still eligible for the study, blood will be collected to measure complete blood counts including haemoglobin for further determination of eligibility.

3. Randomization: A statistician from the Cornell Statistical Consulting Unit will generate the random allocation sequence using a statistical software package (SAS version 9.4); the randomization sequence key will be blinded to all study personnel except the study statistician and the Filemaker database developer until follow-up is over. Randomization will be allocated at the individual level. Individuals will be randomized in blocks of 60. Pearl millet food products will coded by study arm (Arm 1: iron- and zinc-biofortified pearl millet; Arm 2: conventional pearl millet). Children will be randomized to either study arm and will be monitored to ascertain they consume the assigned food product throughout the study duration. The midline measurement (including biological sample collection) will be a random serial sample

For peer review only

taken at any month (months 2-7) between the baseline and endline measurements[15]. Longitudinal midline random serial sampling is often used in population pharmacokinetic research and has been shown to be a useful strategy for iron fortification efficacy studies, by describing the pattern of iron repletion[15].

Intervention and comparator

The intervention is iron-and-zinc-biofortified pearl millet (ICTP-8203) developed by the International Crops Research Institute for the Semi-Arid Tropics (ICRISAT), and the comparator is a conventional pearl millet that is commercially available on the market. The comparator was chosen because it is similar to the intervention in all aspects, except for iron and zinc content, which allows direct analysis of the impact of the iron-and-zinc-biofortified variety on our outcome measurements. The nutrient composition of biofortified and the conventional pearl millet flour are presented in Table 1. We expect a child to consume an average of 60 g of pearl millet per day, depending on the age of the child[11].

Table 1: Nutrient composition of pearl millet varieties

	Biofortified (per 100 g flour)	Control (per 100 g flour)
Moisture (g)	6.89	7.16
Fat (g)	7.88	8.74
Protein (g)	13.46	13.46
Carbohydrate (g)	30.22	30.98
Energy (Kcal)	246	256
Ash (g)	1.99	1.63
Phytate (mg)	876.59	998.44
Iron (mg)	6.64	2.56
Zinc (mg)	4.43	1.24

For peer review only

Inclusion and exclusion criteria

Inclusion criteria

Participants included in this study will be 12-18-month-old male and female children with haemoglobin concentration greater than or equal to 9 g/dL, living in urban slums of Mumbai.

Exclusion criteria

Children will be excluded if: (1) they are younger than 12 months, 0 days old or older than 18 months, 30 days at enrolment; (2) their haemoglobin concentration is less than 9 g/dL or haemoglobinopathy is present (as indicated via abnormal peak via hemoglobin variant analysis and confirmed by CSSC physicians); (3) they show signs of severe malnutrition (a weight-for-length -3)[16]; (4) their caregiver reports prior known diagnosis of HIV, tuberculosis, or current diagnoses of HIV, malaria, tuberculosis, and/or dengue fever; (5) their caregiver is unavailable to bring the child to the feeding centre during follow-up; (6) the child has any known dietary allergies; and (7) their caregivers will leave the study site for greater than 4 weeks during the follow-up period; (8) prior (within the past one year) or current consumption of iron or zinc supplements. Children who are severely anemic will be referred to physicians at the Center for the Study of Social Change (CSSC).

Informed consent process

Research assistants will obtain informed consent from caregivers. Three consent forms will be collected at the Screening Visit: 1) Pre-Screening Consent (non-invasive questionnaires to determine age eligibility, use of dietary supplements, availability of caregiver, etc. as described in Exclusion Criteria above; 2) Screening Consent (invasive procedures including blood collection and anthropometric

For peer review only

measurements to assess malnutrition); and 3) Intervention Consent (including baseline procedures such as a full doctor's exam and detailed background questionnaires).

Feeding Centre

Feeding centres will be located near to where children and their caregivers' dwellings are clustered, to ensure that travel time from their home to the feeding centre is within walking distance.

Sample size estimation

Our estimates of sample size are based on assumptions about mean values and associated variation in haemoglobin and serum ferritin. We expect 50–75% of these children to have iron and zinc deficiency based on published literature[17] and preliminary results (unpublished). We assume that absorbed iron will be transferred to body stores in the liver, and that the change in liver stores is reflected in changes in serum ferritin at a rate of 8 μg/L of serum ferritin per mg liver iron in non-anaemic, iron-depleted children. Iron-deficient, anaemic (haemoglobin < 11 g/dL[18]) children will have their absorbable iron directed to haemoglobin synthesis in the early months of the feeding trial. We estimate that the children consuming biofortified FeZn-PM will demonstrate a gain of 1 g/dL in haemoglobin concentrations in 2.4 months, whereas children consuming control pearl millet will need more than 9 months to demonstrate the same increase. To detect a significant increase in ferritin from the baseline value of 1.79 (6 mg/L) with standard deviation of 1.2, in the experimental group, compared to the control group, at a power of 80% and 5% significance level, 96 participants will be required.

For peer review only - http://bmjopen.bmj.com/site/about/guidelines.xhtml

Follow-up

Assessments

At baseline, we will assess anthropometry; collect blood for analysis of hemoglobin, iron, zinc, CRP, and AGP biomarkers in the blood (all blood analyses other than complete blood counts and T cell counts will be stored at -80 and then performed in batch at the end of the trial); nutrient intake measures using multiple-pass 24-h dietary recall[19] for children and mothers; socio-economic and demographic information; and morbidity history. Follow-up will continue for 9 months and will include monthly anthropometric measurements, morbidity assessments, Infant and Young Child Feeding Questionnaire[20], and 24-hour dietary recall[19]. Both maternal and infant dietary data will be collected via paper forms and entered into the CS Dietary System Rel. 1.10, to calculate energy and nutrient intakes. Additionally, we will collect rectal swab or stool samples to determine microbiome composition in potential future ancillary analyses. The midline time point will be a random serial sample that occurs between the baseline and endline measures. Children may visit the CSSC clinic at any time during the trial for healthcare treatment. All blood collection procedures will include the use a local anaesthetic (Prilox- Lidocaine and Prilocaine cream) to decrease pain, and a Vein Finder device to illuminate the veins to better identify the injection site.

Administration of intervention

Both arms will receive complementary foods prepared with pearl millet three times per day, 6 days per week, for 9 months. Culturally acceptable pearl millet based complementary foods were developed by SNDT University and the acceptability of

For peer review only

these food products was tested on the caregivers and the children from the slums of Mumbai[10]. We will conduct a run-in/pilot phase of the study with 1-3 selected feeding centres.

Each day, the child's caregiver will bring the child to their feeding centre. Two meals will be consumed at the feeding centre; the third meal may be consumed at home. The food intake of the two meals at the feeding centre will be measured directly before and after consumption. To measure the intake of the third meal, the weight of unconsumed food from the third meal will be measured the next morning at the feeding centre. To assure adherence, healthcare workers will follow up with the participants' caregivers and record reasons for non-adherence. Throughout the study, we will conduct periodic analysis of random samples of both grain varieties and prepared food products to ensure food safety and quality of the intervention.

Primary outcome measurements

Biomarkers of iron and zinc status

We will determine if biofortified pearl millet improves iron and zinc status compared to children who consume non-biofortified pearl millet. Specifically, iron and zinc status will be assessed by measuring concentrations of haemoglobin (Hb), serum ferritin, serum transferrin receptor (sTfR) and plasma zinc at enrolment (baseline), midline, and endline. Additionally, we will measure concentrations of inflammatory biomarkers C-reactive protein (CRP) and alpha 1-acid glycoprotein (AGP), as iron and zinc biomarkers can be influenced by inflammation.

Growth

For peer review only

We will assess change in a child's growth as another outcome variable to determine if iron-biofortified pearl millet reduces the risk of underweight (weight-for-age z score < -2), wasting (weight-for-height z-score < -2) and stunting (length-for-age z score < -2) during the 9-month intervention period, compared to those who receive non-biofortified pearl millet. We will measure weight (kg) and length (cm) at baseline and monthly throughout follow-up. The weight and length data will be converted to weight-for-age, weight-for-length z-score, and length-for-age z-score. The growth rates (as kg/month, and cm/month) will also be compared from the absolute measurements after controlling for age.

Additional outcomes

Immune function

We will assess immune function by measuring T cell counts and vaccine responses. Additionally, we will collect morbidity data, including changes in types and frequencies of morbidities such as diarrhoeal illness, pneumonia, and any chronic disease throughout follow-up during each clinic visit. Caregivers will also be encouraged to come to the clinic at any time for any health-related reasons, which will also be recorded. This will provide an accurate representation of both innate and adaptive immunity in participants.

Cognitive function

To determine if consumption of biofortified pearl millet improves child cognitive function, compared to consumption of non-biofortified pearl millet, we will assess in a subset of children aspects of cognition that (1) previously have been shown to be sensitive to the effects of early iron and/or zinc deficiency, or (2) draw heavily upon

For peer review only

brain structures or processes thought to be vulnerable to early iron and/or zinc deficiency, based on studies of animal models and humans. Thus, we will assess multiple specific aspects of memory, attention and processing speed using automated eye trackers[21]. In addition, we will also assess higher-level, integrative cognitive abilities, including problem-solving and exploratory behavior and global aspects of attention and inattention during free play with toys[22].

Data collection and storage

Prior to data collection, the field staff will be trained on ethical and data collection procedures. The protocol of data collection forms to be used is shown in Figure 1. All data except dietary data will be collected on iPads or laptops for the proposed project. We will use a mobile electronic data capture (mEDC) framework on the iOS platform, *Connedct,* specifically designed for this project[23]. A secure server will be utilized for uploading, storing, and accessing the data. This will enable real-time feedback and error checking, as well as eliminate errors associated with data entry, facilitating faster data analysis and rapid dissemination of results. All information will be kept confidential. All names will be removed from the data for analysis. The identifying codes and linked names will be securely stored in password-protected computers. All data will be securely stored on a third-party server, which will have limited access by study team members. All biological specimens will be collected by trained medical professionals and evaluated by certified laboratories within India. Biological specimens will be stored appropriately throughout the duration of the study and after the study for future analysis.

Data analysis

For peer review only

We will use an intention-to-treat approach to determine the effect of biofortified pearl millet on the outcomes described above. Advanced analysis will use mixed models to account for pearl millet-based complementary foods consumed and use each block of 60 as a random effect. We will also plot dose-response curves using restricted cubic splines that will help us detect any threshold effects, as well as non-linear associations. Nonparametric tests, such as the Hodges-Lehmann test, will be used where relevant, as in the case of non-normally distributed variables including serum ferritin.

Study monitoring board

A Data Safety Monitoring Board (DSMB) will be established for this study and will include experts representing Cornell University and SNDT University who are not directly involved in the trial. The DSMB members will oversee the study and will periodically monitor the progress and outcomes of the intervention.

Reporting of adverse events

All adverse events will be reported by the study physician via an Adverse Event/Serious Adverse Event Report (AE/SAE) form. When an adverse event occurs, study physicians, research assistants and/or other study personnel will undertake all necessary precautions to ensure the safety and well-being of the participant.

The following study protocol endpoints will be considered to define safety and efficacy outcomes, and establish un-blinding and stopping guidelines in this trial, as per the discretion of the DSMB: 1. Diagnosis of the development of severe acute

For peer review only - http://bmjopen.bmj.com/site/about/guidelines.xhtml

malnutrition such as Kwashiorkor; 2. Occurrence of all-cause death. Demonstration of efficacy, namely a significant beneficial effect on mortality and other adverse outcomes will be used as a guideline to determine if the study should be un-blinded, stopped, or terminated.

ETHICS AND DISSEMINATION

Before conducting interviews or allowing participation in the study, written informed consent will be obtained from each participant's guardians/primary caregivers, and research assistants will record each granting of informed consent using audio and video technology. Severely anaemic (<7 g/dL) and malnourished children will be provided with appropriate medical care and referred to CSSC. The caregivers will be assured of the confidentiality and anonymity of reports and publications generated from this study. Participation will be voluntary, and participants will be assured that refusal to participate in the study will not impact their access to care. The ethical clearance for this study has been obtained from the Institutional Review Board of Cornell University; Intersystem Biomedical Ethics Committee (ISBEC) of SNDT University, Mumbai, India; and the Institutional Review Board of St. John's Research Institute, Bangalore, India. This study has received clearance from Health Ministry's Screening Committee (HMSC) of the ICMR. The results of this study will be disseminated at several research conferences and as published articles in peer-reviewed journals. The present study protocol was prepared in accordance to the SPIRIT statement[24]. This trial has been registered at Clinicaltrials.gov (registration number NCT02233764) and the Clinical Trials Registry of India (CTRI) (reference number REF/2014/10/007731, CTRI number CTRI/2015/11/006376).

For peer review only

REFERENCES

1. Black MM, Quigg AM, Hurley KM, et al. Iron deficiency and iron-deficiency anemia in the first two years of life: strategies to prevent loss of developmental potential. *Nutrition reviews* 2011;69 Suppl 1:S64-70. doi: 10.1111/j.1753-4887.2011.00435.x
2. Black RE, Victora CG, Walker SP, et al. Maternal and child undernutrition and overweight in low-income and middle-income countries. *The Lancet* 2013;382(9890):427-51. doi: 10.1016/s0140-6736(13)60937-x
3. Vucic V, Berti C, Vollhardt C, et al. Effect of iron intervention on growth during gestation, infancy, childhood, and adolescence: a systematic review with meta-analysis. *Nutrition reviews* 2013;71(6):386-401. doi: 10.1111/nure.12037
4. Lawless J, Latham M, Kinoti S, et al. Iron Supplementation Improves Appetite and Growth in Anemic Kenyan Primary School Children. *Journal of Nutrition* 1994;124:645-54.
5. Kanani S, RH P. Symposium Supplementation with Iron and Folic Acid Enhances Growth in Adolescent Indian Girls. *Journal of Nutrition* 2000;130(452S-455S)
6. Cole CR, Lifshitz F. Zinc nutrition and growth retardation. *Pediatr Endocrinol Rev* 2008;5(4):889-96.
7. Walter T, Olivares M, Pizarro F, et al. Iron, anemia, and infection. *Nutrition reviews* 1997;55(4):111-24.
8. Georgieff MK. Long-term brain and behavioral consequences of early iron deficiency. *Nutrition reviews* 2011;69 Suppl 1:S43-8. doi: 10.1111/j.1753-4887.2011.00432.x
9. HE B. Plant Breeding- A New Tool for Fighting Micronutrient Malnutrition. *Am Soc Nutr Sci* 2002:511-3.
10. Huey SL, Venkatramanan S, Udipi SA, et al. Acceptability of Iron- and Zinc-Biofortified Pearl Millet (ICTP-8203)-based Complementary Foods Among Children in an Urban Slum of Mumbai, India. *Frontiers in Nutrition* 2017;4(August 2017, Article 39):1-10. doi: 10.3389/fnut.2017.00039
11. Acceptability of iron biofortified pearl millet among young children in urban slums of Mumbai, India. Experimental Biology; 2016; San Diego. FASEB journal : official publication of the Federation of American Societies for Experimental Biology.
12. Kodkany BS, Bellad RM, Mahantshetti NS, et al. Biofortification of pearl millet with iron and zinc in a randomized controlled trial increases absorption of these minerals above physiologic requirements in young children. *The Journal of nutrition* 2013;143(9):1489-93. doi: 10.3945/jn.113.176677
13. I.C.M.R. Nutrient Requirements and Recommended Dietary Allowances for Indians. In: Research EGotICoM, ed. Hyderabad, India: National Institute of Nutrition, Indian Council of Medical Research, 2009.
14. Chandramouli C. HOUSING STOCK, AMENITIES & ASSETS IN SLUMS - CENSUS 2011 (Presentation). In: Affairs MoH, ed. India: Census of India, 2011.

For peer review only

15. Andersson M, Theis W, Zimmermann MB, et al. Random serial sampling to evaluate efficacy of iron fortification: a randomized controlled trial of margarine fortification with ferric pyrophosphate or sodium iron edetate. *The American journal of clinical nutrition* 2010;92(5):1094-104. doi: 10.3945/ajcn.2010.29523
16. de Onis M, Onyango A, Borghi E, et al. Worldwide implementation of the WHO Child Growth Standards. *Public health nutrition* 2012;15(9):1603-10. doi: 10.1017/S136898001200105X
17. Kapoor D, Agarwal KN, Sharma S, et al. Iron status of children aged 9-36 months in an urban slum Integrated Child Development Services project in Delhi. *Indian Pediatr* 2002;39(2):136-44.
18. WHO/NMH/NHD/11.1. Haemoglobin concentrations for the diagnosis of anaemia and assessment of severity. 2011
19. Rizek RL, Pao EM. Dietary intake methodology I. USDA surveys and supporting research. *The Journal of nutrition* 1990;120 Suppl 11:1525-9.
20. Organization WH. Indicators for assessing infant and young child feeding practices, 2008.
21. Aslin R, B M. Automated Corneal-Reflection Eye Tracking in Infancy- Methodological Developments and Applications to Cognition. *Infancy* 2004;6:155-63. doi: 10.1207/s15327078in0602_1
22. Ruff HA, Lawson KR, Parrinello R, et al. Long-term stability of individual differences in sustained attention in the early years. *Child Dev* 1990;61(1):60-75.
23. Ruth C, Yu EA, Huey SL, et al. ConnEDCt: A Development Framework for Mobile Electronic Data Capture in Disconnected Communities. *Computer Science and Education in Computer Science* 2016;12:219-33.
24. Chan A-W, Tetzlaff J, Altman D, et al. SPIRIT 2013 Statement- Defining Standard Protocol Items for Clinical Trials. *Ann Intern Med* 2013;158:200-7.

AUTHORS' CONTRIBUTIONS: Saurabh Mehta, Julia L. Finkelstein and Jere D. Haas designed the study, conceived the research questions and prepared the study protocol. Saurabh Mehta is the principal investigator of the study and Julia L. Finkelstein, Jere D. Haas, Shobha A. Udipi, Padmini Ghugre, Richard L. Canfield, Anura V. Kurpad, and Ramesh D. Potdar are co-investigators. Caleb Ruth developed the database used in this trial and provided feedback on the protocol. Samantha L. Huey and Sudha Venkatramanan contributed to the editing of the protocol and will supervise the data collection under the guidance of the investigators. All authors read and approved the final protocol.

For peer review only

ABBREVIATIONS: AGP: Alpha 1-acid glycoprotein; CRP: C-reactive protein; Fe: Iron; FeZn-PM: iron- and zinc-enhanced variety of pearl millet; ICRISAT: International Crops Research Institute for the Semi-Arid Tropics; ICTP8203-Fe: high iron and zinc-biofortified pearl millet variety; ICMR: Indian Council of Medical Research; ISBEC: Intersystem Biomedical Ethics Committee; IRB: Institutional Review Board; KHS: Kasturba Health Society; PM: Pearl Millet; RDA: Recommended Daily Allowance; SJRI: St. John's Research Institute; SNDT: Shreemati Nathibai Damodar Thackersey; sTfR: Serum transferrin receptor; Zn: Zinc

FUNDING: This work was supported by HarvestPlus, grant number 2014H8302.
Address:
HarvestPlus
2033 K St NW
Washington, DC 20006

CONFLICTS OF INTEREST: SM is an unpaid board member for a diagnostic start up focused on developing point-of-care assays for nutritional status informed by his research as a faculty member at Cornell University. All other authors report no conflict of interest.

Figure Legends:

Figure 1. Form collection protocol.

For peer review only

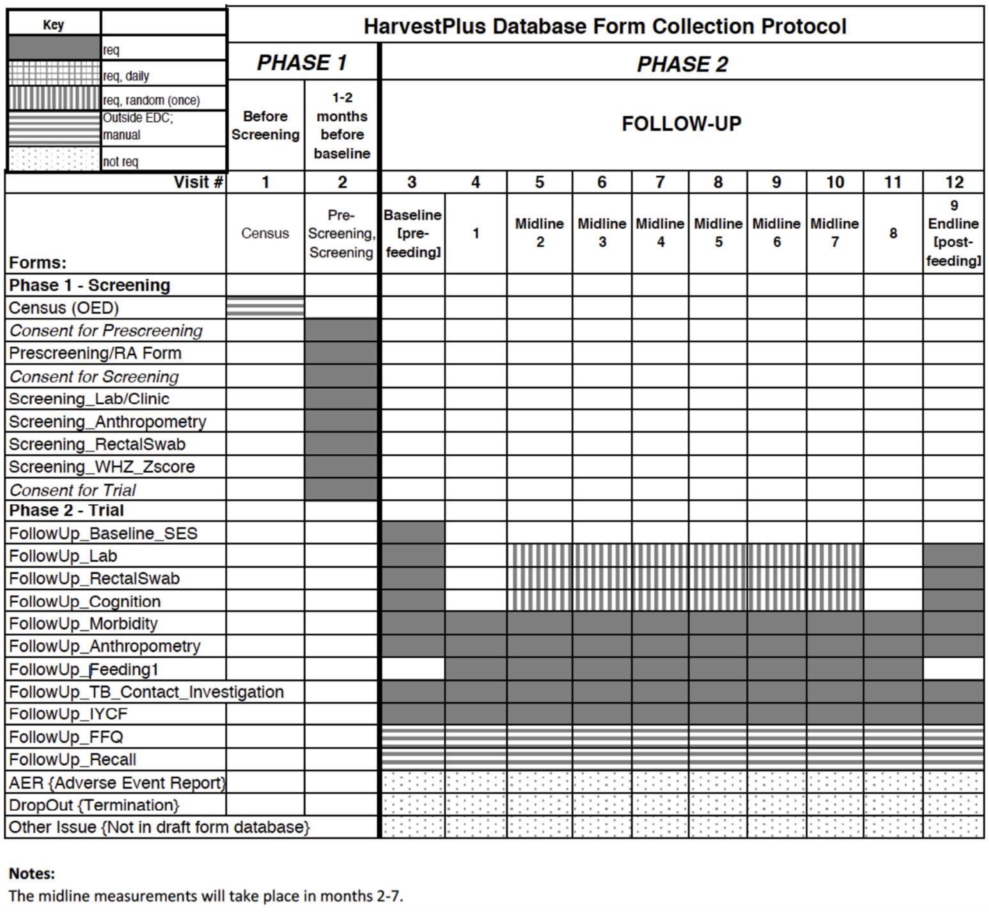

Figure 1. Form collection protocol.

159x146mm (300 x 300 DPI)

only

SPIRIT 2013 Checklist: Recommended items to address in a clinical trial protocol and related documents*

Section/item	Item No	Description	Addressed on page number
**Administrative information**			
Title	1	Descriptive title identifying the study design, population, interventions, and, if applicable, trial acronym	_1____________
Trial registration	2a	Trial identifier and registry name. If not yet registered, name of intended registry	_3,16__________
	2b	All items from the World Health Organization Trial Registration Data Set	_yes__________
Protocol version	3	Date and version identifier	_N/A_________
Funding	4	Sources and types of financial, material, and other support	_19
Roles and responsibilities	5a	Names, affiliations, and roles of protocol contributors	_1, 18_________
	5b	Name and contact information for the trial sponsor	_19____________
	5c	Role of study sponsor and funders, if any, in study design; collection, management, analysis, and interpretation of data; writing of the report; and the decision to submit the report for publication, including whether they will have ultimate authority over any of these activities	_18____________
	5d	Composition, roles, and responsibilities of the coordinating centre, steering committee, endpoint adjudication committee, data management team, and other individuals or groups overseeing the trial, if applicable (see Item 21a for data monitoring committee)	_15____________

For peer review only - http://bmjopen.bmj.com/site/about/guidelines.xhtml

**Introduction**			
Background and rationale	6a	Description of research question and justification for undertaking the trial, including summary of relevant studies (published and unpublished) examining benefits and harms for each intervention	_5, 6_________
	6b	Explanation for choice of comparators	_7, 8___________
Objectives	7	Specific objectives or hypotheses	_6_____________
Trial design	8	Description of trial design including type of trial (eg, parallel group, crossover, factorial, single group), allocation ratio, and framework (eg, superiority, equivalence, noninferiority, exploratory)	_6___________
**Methods: Participants, interventions, and outcomes**			
Study setting	9	Description of study settings (eg, community clinic, academic hospital) and list of countries where data will be collected. Reference to where list of study sites can be obtained	_6_____________
Eligibility criteria	10	Inclusion and exclusion criteria for participants. If applicable, eligibility criteria for study centres and individuals who will perform the interventions (eg, surgeons, psychotherapists)	__8,9______
Interventions	11a	Interventions for each group with sufficient detail to allow replication, including how and when they will be administered	_9,10__________
	11b	Criteria for discontinuing or modifying allocated interventions for a given trial participant (eg, drug dose change in response to harms, participant request, or improving/worsening disease)	_11____________
	11c	Strategies to improve adherence to intervention protocols, and any procedures for monitoring adherence (eg, drug tablet return, laboratory tests)	_12____________
	11d	Relevant concomitant care and interventions that are permitted or prohibited during the trial	_10,11_________
Outcomes	12	Primary, secondary, and other outcomes, including the specific measurement variable (eg, systolic blood pressure), analysis metric (eg, change from baseline, final value, time to event), method of aggregation (eg, median, proportion), and time point for each outcome. Explanation of the clinical relevance of chosen efficacy and harm outcomes is strongly recommended	_11-13________
Participant timeline	13	Time schedule of enrolment, interventions (including any run-ins and washouts), assessments, and visits for participants. A schematic diagram is highly recommended (see Figure)	_x (see attached figure) __

For peer review only - http://bmjopen.bmj.com/site/about/guidelines.xhtml

Sample size	14	Estimated number of participants needed to achieve study objectives and how it was determined, including clinical and statistical assumptions supporting any sample size calculations	__10,11_______
Recruitment	15	Strategies for achieving adequate participant enrolment to reach target sample size	_10,11______

Methods: Assignment of interventions (for controlled trials)

Allocation:

Sequence generation	16a	Method of generating the allocation sequence (eg, computer-generated random numbers), and list of any factors for stratification. To reduce predictability of a random sequence, details of any planned restriction (eg, blocking) should be provided in a separate document that is unavailable to those who enrol participants or assign interventions	_7________
Allocation concealment mechanism	16b	Mechanism of implementing the allocation sequence (eg, central telephone; sequentially numbered, opaque, sealed envelopes), describing any steps to conceal the sequence until interventions are assigned	_7,8______
Implementation	16c	Who will generate the allocation sequence, who will enrol participants, and who will assign participants to interventions	_7____________
Blinding (masking)	17a	Who will be blinded after assignment to interventions (eg, trial participants, care providers, outcome assessors, data analysts), and how	_4,7__________
	17b	If blinded, circumstances under which unblinding is permissible, and procedure for revealing a participant's allocated intervention during the trial	_N/A________

Methods: Data collection, management, and analysis

Data collection methods	18a	Plans for assessment and collection of outcome, baseline, and other trial data, including any related processes to promote data quality (eg, duplicate measurements, training of assessors) and a description of study instruments (eg, questionnaires, laboratory tests) along with their reliability and validity, if known. Reference to where data collection forms can be found, if not in the protocol	_12-14________
	18b	Plans to promote participant retention and complete follow-up, including list of any outcome data to be collected for participants who discontinue or deviate from intervention protocols	_12, 16_____

For peer review only - http://bmjopen.bmj.com/site/about/guidelines.xhtml

Data management	19	Plans for data entry, coding, security, and storage, including any related processes to promote data quality (eg, double data entry; range checks for data values). Reference to where details of data management procedures can be found, if not in the protocol	_14____________
Statistical methods	20a	Statistical methods for analysing primary and secondary outcomes. Reference to where other details of the statistical analysis plan can be found, if not in the protocol	_15____________
	20b	Methods for any additional analyses (eg, subgroup and adjusted analyses)	_15____________
	20c	Definition of analysis population relating to protocol non-adherence (eg, as randomised analysis), and any statistical methods to handle missing data (eg, multiple imputation)	_15____________
**Methods: Monitoring**			
Data monitoring	21a	Composition of data monitoring committee (DMC); summary of its role and reporting structure; statement of whether it is independent from the sponsor and competing interests; and reference to where further details about its charter can be found, if not in the protocol. Alternatively, an explanation of why a DMC is not needed	_15_________
	21b	Description of any interim analyses and stopping guidelines, including who will have access to these interim results and make the final decision to terminate the trial	_16________
Harms	22	Plans for collecting, assessing, reporting, and managing solicited and spontaneously reported adverse events and other unintended effects of trial interventions or trial conduct	_15,16________
Auditing	23	Frequency and procedures for auditing trial conduct, if any, and whether the process will be independent from investigators and the sponsor	_15_________
**Ethics and dissemination**			
Research ethics approval	24	Plans for seeking research ethics committee/institutional review board (REC/IRB) approval	_16,17________
Protocol amendments	25	Plans for communicating important protocol modifications (eg, changes to eligibility criteria, outcomes, analyses) to relevant parties (eg, investigators, REC/IRBs, trial participants, trial registries, journals, regulators)	_16,17_______

For peer review only - http://bmjopen.bmj.com/site/about/guidelines.xhtml

Consent or assent	26a	Who will obtain informed consent or assent from potential trial participants or authorised surrogates, and how (see Item 32)	_9,10________
	26b	Additional consent provisions for collection and use of participant data and biological specimens in ancillary studies, if applicable	_9,10 _
Confidentiality	27	How personal information about potential and enrolled participants will be collected, shared, and maintained in order to protect confidentiality before, during, and after the trial	_14,15__
Declaration of interests	28	Financial and other competing interests for principal investigators for the overall trial and each study site	_20____________
Access to data	29	Statement of who will have access to the final trial dataset, and disclosure of contractual agreements that limit such access for investigators	_15____________
Ancillary and post-trial care	30	Provisions, if any, for ancillary and post-trial care, and for compensation to those who suffer harm from trial participation	_16____________
Dissemination policy	31a	Plans for investigators and sponsor to communicate trial results to participants, healthcare professionals, the public, and other relevant groups (eg, via publication, reporting in results databases, or other data sharing arrangements), including any publication restrictions	_16,17_________
	31b	Authorship eligibility guidelines and any intended use of professional writers	_N/A___________
	31c	Plans, if any, for granting public access to the full protocol, participant-level dataset, and statistical code	_17___________
**Appendices**			
Informed consent materials	32	Model consent form and other related documentation given to participants and authorised surrogates	_attached___
Biological specimens	33	Plans for collection, laboratory evaluation, and storage of biological specimens for genetic or molecular analysis in the current trial and for future use in ancillary studies, if applicable	__15______

*It is strongly recommended that this checklist be read in conjunction with the SPIRIT 2013 Explanation & Elaboration for important clarification on the items. Amendments to the protocol should be tracked and dated. The SPIRIT checklist is copyrighted by the SPIRIT Group under the Creative Commons "Attribution-NonCommercial-NoDerivs 3.0 Unported" license.

For peer review only - http://bmjopen.bmj.com/site/about/guidelines.xhtml
